# Assessing the value of human papillomavirus vaccination in Gavi-eligible low-income and middle-income countries

**DOI:** 10.1136/bmjgh-2020-003006

**Published:** 2020-10-20

**Authors:** Jessica Ochalek, Kaja Abbas, Karl Claxton, Mark Jit, James Lomas

**Affiliations:** 1 Centre for Health Economics, University of York, York, United Kingdom; 2 Department of Infectious Disease Epidemiology, Faculty of Epidemiology and Population Health, London School of Hygiene & Tropical Medicine, London, United Kingdom; 3 Modelling and Economics Unit, Public Health England, London, United Kingdom; 4 School of Public Health, University of Hong Kong, Hong Kong Special Administrative Region, China

**Keywords:** health economics, vaccines

## Abstract

**Introduction:**

Estimating the value of providing effective healthcare interventions in a country requires an assessment of whether the improvement in health outcomes they offer exceeds the improvement in health that would have been possible if the resources required had, instead, been made available for other healthcare activities in that country. This potential alternative use of the same resources represents the health opportunity cost of providing the intervention. Without such assessments, there is a danger that blanket recommendations made by international organisations will lead to the adoption of healthcare interventions that are not cost effective in some countries, even given existing donor mechanisms intended to support their affordability.

**Methods:**

We assessed the net health impact to 46 Gavi-eligible countries of achieving one of the WHO’s proposed 90-70-90 targets for cervical cancer elimination, which includes 90% coverage of human papillomavirus (HPV) vaccination among girls by 15 years of age, using published estimates of the expected additional benefits and costs in each country and estimates of the marginal productivity of each healthcare system. We calculated the maximum price each country could afford to pay for HPV vaccination to be cost effective by assessing the net health impact that would be expected to be generated at different potential prices.

**Results:**

At Gavi negotiated prices, HPV vaccination offers net health benefits across most Gavi-eligible countries included in this study. However, if Gavi-eligible countries faced the average price faced by non-Gavi eligible countries, providing HPV vaccination would result in reduced overall population health in most countries.

**Conclusion:**

Estimates of the net health impact of providing a healthcare intervention can be used to assess the benefit (or lack of) to countries of adhering to global guidance, inform negotiations with donors, as well as pricing negotiations and the value of developing new healthcare interventions.

**Key questions**

**What is already known?**

- Human papillomavirus (HPV) vaccination is considered cost effective in almost every country when compared against a threshold of 1× gross domestic product (GDP) per capita.
- GDP per capita-based thresholds are no longer recommended for judging the value for money of healthcare interventions.
- Country-specific health opportunity costs enable the estimation of the impact of an intervention in terms of the population net health benefits.

**What are the new findings?**

- Health opportunity costs in Gavi-eligible low-income and middle-income countries can be used to estimate the scale of the expected net impact on population health of HPV vaccination.
- At Gavi negotiated prices, HPV vaccination offers positive net health benefits in most of the Gavi-eligible countries included in this study.
- If Gavi-eligible countries faced the same price as non-Gavi eligible countries, providing HPV vaccination would improve overall population health in 13 countries and reduce overall population health in 33 countries imposing a net burden of 38 million disability-adjusted life years.

**What do the new findings imply?**

- Gavi’s negotiations on behalf of countries eligible for its support have succeeded in making adhering to the WHO guidance around HPV vaccination a beneficial aim for most countries.
- Determining prices using these methods, which account for country-specific health opportunity costs, offers an opportunity to ensure that all countries can benefit from adopting HPV vaccination or other recommendations made by global bodies.
- Assessing the likely scale and distribution of the impact of potential new interventions on net population health, at a particular price, are valuable for informing priorities in developing new healthcare interventions.

## Introduction

In 2019, donors funded 27% of healthcare provided in low-income countries and 3% in lower middle income countries.^1^ How donors make decisions around which interventions to fund is unclear.^2^ Funding may be tied to a donor’s strategic mission or aligned to international guidance or recommendations. Estimating the value of providing effective interventions in a country—including vaccines, drugs, and diagnostics, as well as prioritising the development of new ones—requires an assessment of whether the improvement in health outcomes they offer exceeds the improvement in health that would have been possible if the resources required had, instead, been made available for other healthcare activities in that country. This potential alternative use of the same resources represents the health opportunity cost of providing the intervention. Without such assessments, there is a danger that blanket recommendations made by international organisations will lead to the adoption of healthcare interventions that are not cost effective in some countries, even given existing donor mechanisms intended to support their affordability.

To assess the health opportunity cost of providing an intervention in a given country, an assessment of the health effects if the additional resources required had, instead, been made available to other healthcare activities is needed. This requires country-specific estimates of the health effects of changes in healthcare expenditure. Such estimates are now available for a limited number of high-income countries based on within-country data and a wider range of high-income as well as low-/middle-income countries (LMICs) based on country-level data. These are typically reported as a cost per quality-adjusted life year (QALY) gained or disability-adjusted life years (DALY) averted.^3–13^

Comparing the additional cost per QALY gained or DALY averted by an intervention with an estimate of cost per QALY gained or DALY averted that reflects health opportunity costs enables a binary assessment of whether the intervention produces health at a better (worse) rate than interventions already funded by the healthcare system (HCS)—that is, is below (above) the estimate of cost per QALY gained or DALY averted that reflects health opportunity costs. However, binary assessments such as these obscure valuable information about the scale of the net benefits (or losses) associated with providing or developing an intervention, and other local context-specific important criteria of affordability, budget impact, fairness and feasibility.^14^ This is particularly important when assessments of value are made across countries; an intervention may be expected to generate a net benefit in some, but a net loss in others.

Explicit consideration of the trade-offs between, for example, providing an intervention in all versus some countries requires quantifying the scale of the benefits (or losses) across countries. The scale of potential net benefits (or losses) associated with providing an intervention in a country can be measured by the net health impact of the intervention: that is the health that is generated by it minus its health opportunity cost. The health opportunity costs associated with additional healthcare expenditure in each HCS differs depending on for example, the budget for healthcare, efficiency of current spending, demographic structure and epidemiology. As such, the health opportunity cost of providing the same intervention at the same price, even to the same sized population, will be different in different HCS.

To illustrate this, we assess the net health impact to countries of achieving one of the WHO’s proposed 90-70-90 targets for cervical cancer elimination, which includes 90% coverage of human papillomavirus (HPV) vaccination among girls by 15 years of age.^15^ This paper shows how estimates of the expected additional benefits and costs of HPV vaccination and health opportunity costs can be used to assess the expected net health impact for each country and across countries associated with adhering to the WHO guidance. We also show how these metrics can inform pricing negotiations (eg, between Gavi, the Vaccine Alliance (hereafter Gavi) and manufacturers), and discuss how they may be used to inform the development of new healthcare interventions.

## Methods

Assessing the expected net health impact for each country and across countries requires data on the additional costs and benefits of HPV vaccination for each country and an estimate of the health opportunity costs faced by each countries’ HCS. We consider countries that were Gavi eligible in 2019 (the most recent year for which a list of eligible countries is publicly available).^16^ Estimates of the additional health benefits of HPV vaccination over the period 2020–2029 come from the Papillomavirus Rapid Interface for Modelling and Economics (PRIME) model developed by Jit *et al* (2014) and recently updated by Abbas *et al* (2020).^17 18^ PRIME assumes lifelong vaccine protection, no indirect (herd) effects and no changes to screening programmes.

The additional (ie, net) cost of vaccination ($\Delta C_{i}$) is made up of the cost of procurement ($\Delta C_{i}^{P}$), which is a function of the per dose procurement price and number of doses, the cost of delivering the vaccine in a country ($\Delta C_{i}^{D}$), and the cost savings that result from cervical cancers averted ($\Delta C_{i}^{C}$)

(1) $$\Delta C_{i}=\Delta C_{i}^{p}+\Delta C_{i}^{D}-\Delta C_{i}^{C}$$

The market price for HPV vaccine doses in countries not eligible for Gavi support is on average US$25 per dose.^19^ Below private market rate procurement prices were negotiated by Gavi with HPV vaccine manufacturers, enabling Gavi eligible countries to purchase vaccines through United Nations organisations at US$4.50 per dose.^20 21^ Countries purchase a share of the vaccines provided while Gavi purchases the remainder. The share funded by Gavi is based on the Gavi cofinancing mechanism depending on the funding phase the country is in.^22^ We assume two doses per vaccinated girl in line with the WHO recommended schedule for HPV vaccination, and that the per dose price remains constant in real terms over the period 2020–2029.^23^ Delivery costs are assumed to be US$1.76 per dose (2019 US$) for low-income countries and US$3.87 per dose (2019 US$) for middle-income countries^24^ (originally in 2013 US$, scaled up using gross domestic product (GDP) deflator 2011 Q1 to 2019 Q3 for USA from the US Federal Reserve https://fred.stlouisfed.org/series/GDPDEF). In combination with published estimates of the health opportunity costs faced by different HCS from Ochalek *et al* (2018), the scale of the net health impact of HPV vaccination by HCS, measured in DALYs averted (net DALYs averted, ${NDA}_{i}$, where the i subscript denotes each HCS) can be estimated for each HCS. No estimates of the health opportunity costs associated with additional healthcare expenditure are available for 12 countries, limiting our analysis to 46 Gavi-eligible countries.

(2) $$NDA_{i}=\Delta DALYs_{i}-\frac{\Delta C_{i}}{k_{i}}$$

Net DALYs averted (NDA) for a given HCS is the difference between DALYs averted by an intervention (${∆DALYs}_{i}$) and DALYs that could have been averted with the additional HCS resources required to implement it ($\frac{\Delta C_{i}}{k_{i}}$) (net of any additional cost savings), where $k_{i}$ is the country-specific estimate of health opportunity cost to avert a single DALY. Note that if the net effect of the intervention saves HCS costs, that is, $\Delta C_{i}<0$, then the net DALYs averted is the DALYs averted by the intervention plus the additional DALYs that can also be averted with the cost savings offered.

The scale of the value of the impact can also be reported in terms of the amount of additional healthcare resources which would be required to deliver similar net health impacts (net dollar value, ${N\$V}_{i}$).

(3) $$N\$V_{i}=k_{i}*\Delta DALYs_{i}-\Delta C_{i}$$

The aggregate net effects of providing the HPV vaccine in a group of countries (eg, all countries in a given income category) can be calculated by summing the estimated net health impact or net dollar value by HCS. For example, where HPV is provided in a group, g, of N HCS, these can be calculated as follows:

(4) $$NDA_{g}=\sum_{i=1}^{N}\Delta DALYs_{i}-\frac{\Delta C_{i}}{k_{i}}$$

(5) $$N\$V_{g}=\sum_{i=1}^{N}k_{i}*\Delta DALYs_{i}-\Delta C_{i}$$

An estimate of the health opportunity costs faced by the HCS also enables the calculation of the maximum per dose procurement price, which is calculated by dividing the maximum procurement cost (${{∆C^{P}}_{i}}^{*}$) by the number of required doses (two doses are required for each person in the cohort), that each HCS could afford to pay for HPV vaccine to ensure that the health lost from the money required to fund it is not greater than the benefit it offers (ie, the cost at which the net dollar value to the HCS would be zero).

(6) $$\Delta C_{i}^{P^{*}}=\Delta DALYs_{i}*k_{i}-\Delta C_{i}^{D}+\Delta C_{i}^{C}$$

This can be used to inform pricing negotiations between Gavi and manufacturers in a way that ensures that global access could be provided with no net losses for any HCS.

We illustrate how these assessments can be used to inform global guidance or recommendations and pricing negotiations by assessing four potential policy options:

1. Achieving the WHO recommendation of 90% coverage of HPV vaccination in Gavi-eligible countries at the average market per dose procurement prices (US$25 per dose).
2. Informing country-specific per dose procurement prices that would ensure that HPV vaccination generates a net health benefit in each Gavi-eligible country.
3. Informing per dose procurement prices for country groups (ie, low-income and lower middle income) that would ensure that HPV vaccination generates a net health benefit in each Gavi-eligible country.
4. Achieving the WHO recommendation of 90% coverage of HPV vaccination in all Gavi-eligible countries at current Gavi-negotiated per dose procurement prices (US$4.50 per dose) first assuming current levels of support for procurement (option 4a) and second, assuming no procurement support (option 4b).

Policy option 1 reflects the implementation of blanket recommendations for providing an intervention for an entire set of countries (see, eg, World Health Organization, 2020).^25^ Policy option 4 reflects the practice of negotiating prices to support countries in complying with recommendations. Policy options 2 and 3 reflect potential methods for determining prices.

While all Gavi funded countries face the same per dose price (US$4.50) for HPV vaccines, most Gavi-funded countries pay for only a portion of the vaccines they purchase while the remainder are funded by Gavi. The share funded by Gavi is based on a cofinancing mechanism, and differs for each country depending on the funding phase the country is in and its per capita income.^22^ In the first instance, we assume current Gavi-negotiated per dose procurement prices and with current levels of support (policy option 4a). Since data on the proportion of vaccines purchased by Gavi are not publicly available, we have calculated them based on the number of years a country has been a Gavi funding recipient and the countries’ gross national income (GNI) per capita in each of those years (see online supplemental appendix 1). Second, we assess this scenario assuming that countries pay 100% of the vaccine procurement costs (ie, US$4.50 per dose; which we term policy option 4b). Delivery costs are the same for each option and are current delivery costs.

10.1136/bmjgh-2020-003006.supp1 Supplementary data

We also undertake sensitivity analyses around discount rates and the estimates of the marginal productivity of HCSs used. Global guidance recommends that where country guidance is lacking either 0% for health benefits and 3% for costs or 3% for both are used as discount rates.^26–28^ Following common practice, our base case for each policy option applies a discount rate of 3% to both costs and benefits,^29 30^ and we assess the results where 0% is applied to health benefits in sensitivity analysis. Our base case uses the central estimate of health opportunity cost for each country from Ochalek *et al*.^31^ We also assess each policy option using the minimum and maximum estimates of cost per DALY for each country from Ochalek *et al* (2018) as a sensitivity analysis.^31^

The total net health impact across all countries for the first policy option, where HPV vaccination is implemented in all countries at current market prices, is calculating by aggregating the NDA for each country from equation 2.

The second policy option entails calculating the maximum price each HCS could afford to pay for HPV vaccination to be a cost-effective use of resources in that HCS if it is not already cost effective at average market price (US$25 per dose). This is calculated by determining the maximum total vaccination procurement cost a country can afford to pay, which is the monetary value of the expected health gains of HPV vaccination net of the difference between the delivery costs and cancer treatment costs averted (as set forth in equation 6).

The third policy option, to set a price by HCS group (eg, income group) rather than by country, is also informed by equation 6, but the lowest maximum price affordable from among a group of countries is applied to all countries in the group.

The fourth policy option reflects the total net health impact of implementing HPV vaccination in all countries at Gavi negotiated prices and with current levels of Gavi support (policy option 4a) or at Gavi negotiated prices without procurement support (policy option 4b) and is calculated by aggregating the NDA for each country from equation 2.

## Results

**Figure 1:**
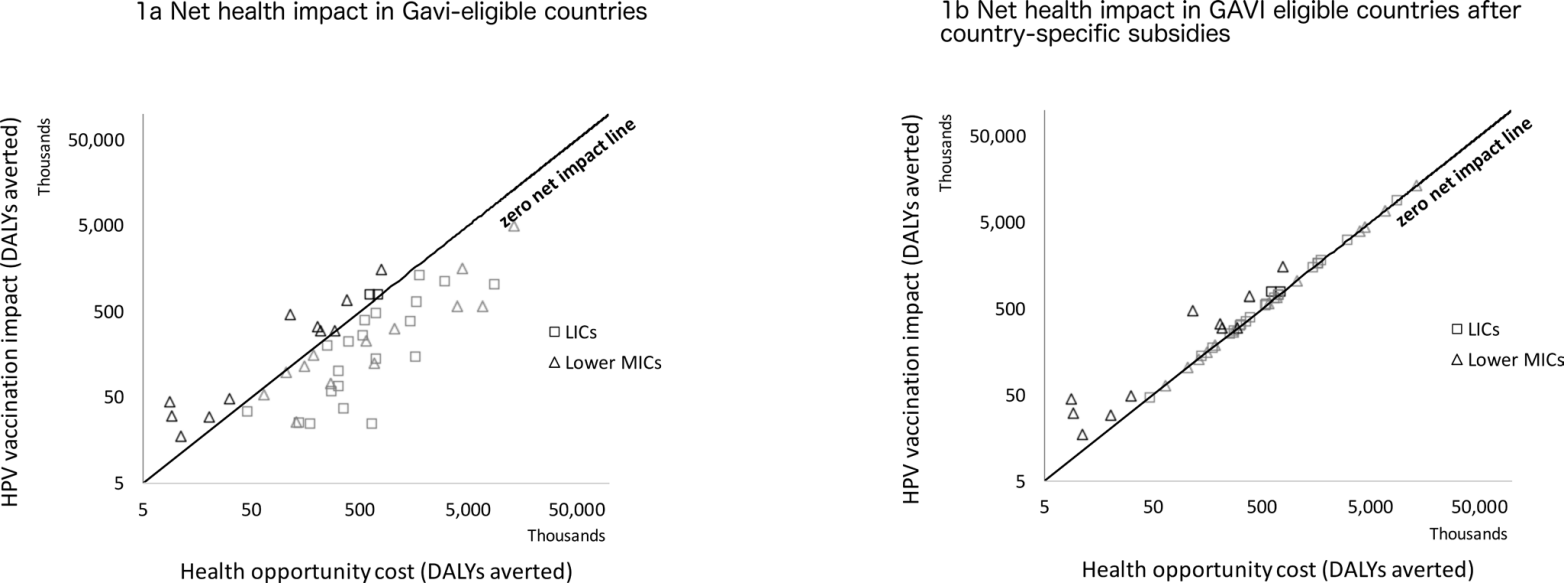
(A) Net health impact in Gavi-eligible countries. (B) Net health impact in Gavi-eligible countries after country-specific subsidies. DALYs, disability-adjusted life years; HPV, human papillomavirus; MICs, middle-income countries.

Figure 1A plots the health gains from the vaccine against the health opportunity cost for each country of achieving the WHO recommendation of 90% coverage of HPV vaccination in all Gavi-eligible countries at average market per dose procurement prices (US$25 per dose) and given current delivery costs (policy option 1). The diagonal line indicates zero net health impact, and (black) points that fall above it refer to countries which have a positive net health impact while (grey) points that fall below it refer to countries which have a negative net health impact.

What is evident is that at this price HPV vaccine results in net health losses in most countries. More countries are below the zero net impact line than above it. The distance between a point above (below) the line and the line is the net health gain (loss) that would result from providing the intervention. There are net health gains in some countries (totalling 2 million DALYs averted); however, these are offset by the net health losses in others (totalling −40 million DALYs averted (see table 1 column 1).

**Table 1:** Net health impact and net monetary impact at three prices

Country	Income category	US$25 per dose (options 1 and 2)		Gavi procurement support (per dose price differs by country, all ≤US$4.50, option 4a)		US$4.50 per dose (option 4b)	
		Net health impact (1000s)	Net monetary impact (1000s 2019 US$)	Net health impact (1000s)	Net monetary impact (1000s 2019 US$)	Net health impact (1000s)	Net monetary impact (1000s 2019 US$)
		(1)	(2)	(3)	(4)	(5)	(6)
Benin	Low income	−222	−47 212	98	20 749	42	8965
Burkina Faso	Low income	−232	−37 900	526	85 735	394	64 298
Burundi	Low income	−164	−19 599	453	54 223	346	41 423
Chad	Low income	−576	−85 115	120	17 705	−1	−123
Democratic Republic of the Congo	Low income	−7746	−495 691	975	62 387	−537	−34 377
Eritrea	Low income	−114	−15 648	21	2854	−3	−354
Ethiopia	Low income	−1944	−397 625	1049	214 497	530	108 363
Gambia	Low income	−12	−3732	35	10 652	27	8158
Guinea	Low income	−276	−36 630	294	39 031	195	25 912
Guinea Bissau	Low income	−151	−9399	27	1701	−4	−223
Haiti	Low income	−216	−38 111	53	9297	6	1077
Madagascar	Low income	−1012	−82 141	822	66 750	504	40 934
Malawi	Low income	163	24 257	896	133 320	769	114 410
Mali	Low income	−1085	−89 221	458	37 691	191	15 686
Mozambique	Low income	38	9024	836	196 248	698	163 786
Nepal	Low income	−172	−46 634	217	58 934	150	40 630
Niger	Low income	−1,505	−157 478	69	7267	−204	−21 298
Rwanda	Low income	−55	−14 362	207	54 440	162	42 511
Sierra Leone	Low income	−320	−39 693	23	2802	−37	−4566
Togo	Low income	−250	−35 745	64	9176	10	1387
Uganda	Low income	−454	−64 882	1523	217 831	1180	168 813
Yemen	Low income	−627	−157 748	−37	−9,388	−125	−31 390
Bangladesh	Lower middle income	−3,406	−504 494	301	44 653	−309	−45 704
Cambodia	Lower middle income	−197	−50 702	63	16 229	20	5079
Cameroon	Lower middle income	−736	−94 408	373	47 898	228	29 285
Comoros	Lower middle income	6	1861	21	6122	19	5565
Côte d'Ivoire	Lower middle income	−346	−85 109	239	58 651	152	37 331
Ghana	Lower middle income	3	1344	276	127 469	276	127 469
India	Lower middle income	−8,392	−2 814 745	2888	968 749	2368	794 116
Kenya	Lower middle income	307	188 020	744	455 613	673	412 218
Kyrgyzstan	Lower middle income	17	14 836	50	43 895	44	38 919
Lesotho	Lower middle income	22	15 009	34	23 484	32	22 375
Mauritania	Lower middle income	-11	-3,685	61	20,443	50	16,865
Nicaragua	Lower middle income	36	97 972	44	118 524	44	118 524
Nigeria	Lower middle income	−2783	−724 904	979	255 030	979	255 030
Pakistan	Lower middle income	−6147	−999 875	−174	−28 328	−1085	−176 560
Republic of Congo	Lower middle income	9	13 848	26	39 162	26	39 162
Republic of Sudan	Lower middle income	−555	−206 489	−42	−15 482	−42	−15 482
Senegal	Lower middle income	79	28 645	335	121 002	300	108 298
South Sudan	Lower middle income	-39	-14 613	122	45,478	96	35,591
Tajikistan	Lower middle income	−104	−43 036	12	4919	−7	−2727
Tanzania	Lower middle income	751	212 282	1989	562 400	1775	502 045
Uzbekistan	Lower middle income	−7	−8595	74	96 739	74	96 739
Vietnam	Lower middle income	−32	−52 974	109	181 915	109	181 915
Zambia	Lower middle income	356	190 274	527	281 374	525	280 238
Zimbabwe	Lower middle income	129	33 316	446	115 670	395	102 315
Low-income countries							
Total net benefits		201	33 281	8766	1 303 289	5203	846 353
Total net losses		−17 134	−1 874 568	−37	−9388	−909	−92 332
Total net impact		−16 932	−1 841 287	8728	1 293 901	4294	754 021
Lower middle income countries							
Total net benefits		1715	797406	9714	3635420	8185	3209078
Total net losses		-22755	-5603628	−216	−43 809	-1442	-240472
Total net impact		−21 041	−4 806222	9498	3 591610	6743	2 968606
All countries							
Total net benefits		1916	830687	18 479	4 938709	13 388	4 055431
Total net losses		−39889	−7 478196	−253	−53 198	−2 351	−3 32803
Total net impact		−3 7973	−6 647509	18 226	4 885511	11036	3 722627

If HPV vaccination were provided only in those countries where it does not reduce overall net health at current average market price, it would be implemented in only 2 of 22 low-income countries and 11 of 24 lower middle income countries where it would generate a net health benefit. While there is a clear benefit to this policy option (ie, it would ensure that overall health across the countries increases as a result of the recommendation, and health is not reduced anywhere), it unlikely to be politically feasible or appealing to restrict access in this way. It may also not be seen as equitable to provide vaccination only to countries that can afford to pay a uniform price for it.

Pricing arrangements that ensure that HPV vaccine generates a net health benefit for each country requires calculating the per dose price at which HPV vaccination would be cost effective in each HCS for which it is not at US$25 per dose (policy option 2). This is visualised in figure 1B, where all of the countries that previously had negative net health impact (as denoted by grey points in figure 1A) are now on the zero net impact line.

Table 2 reports the per dose price at which HPV vaccination would be cost effective in each HCS for which it is not at US$25 per dose. That is the maximum that the country could afford to pay per dose for HPV vaccination to generate zero net benefit (ie, no net loss in overall population health, but also no net benefit). Among countries where a price reduction is required, the price reduction ranges from US$2 to US$26 (2019 US). If the vaccine manufacturer and/or a global donor were to fund the difference for both doses for all eligible girls for each country, it would cost US$9.3 billion (2019 US). The same money could avert 49 million DALYs if spent on existing interventions in these countries instead.

**Table 2:** Country-specific and income group-specific price reductions required

Country	Income group	Per dose price reduction required (2019 US$)	Cohort size (1000s)	Total reduction using country-specific price (1000s, 2019 US$, option 2)	Total reduction using country income group price (1000s, US$−1 for low-income countries, US$0 for lower middle income countries, 2019 US, option 3)
Benin	Low	18	1567	57 749	80 661
Burkina Faso	Low	10	2851	57 074	146 744
Burundi	Low	9	1703	31 075	87 647
Chad	Low	21	2372	101 129	122 104
Democratic Republic of the Congo	Low	23	12 881	582 915	663 039
Eritrea	Low	22	425	18 430	21 877
Ethiopia	Low	17	14 104	491 992	725 995
Gambia	Low	9	332	5981	17 090
Guinea	Low	14	1744	48 351	89 790
Guinea Bissau	Low	22	256	11 104	13 159
Haiti	Low	21	1088	45 173	55 990
Madagascar	Low	15	3434	105 294	176 781
Malawi	Low	0	2510	0	129 186
Mali	Low	19	2927	108 948	150 676
Mozambique	Low	2	4319	20 123	222 326
Nepal	Low	13	2418	62 126	124 454
Niger	Low	24	3811	183 674	196 160
Rwanda	Low	8	1587	25 035	81 666
Sierra Leone	Low	24	978	46 162	50 321
Togo	Low	21	1034	42 623	53 232
Uganda	Low	8	6512	108 355	335 200
Yemen	Low	26	3515	180 923	180 923
Bangladesh	Lower middle	23	12 719	592 799	645 928
Cambodia	Lower middle	20	1549	61 602	78 677
Cameroon	Lower middle	17	3447	119 244	175 050
Comoros	Lower middle	0	103	0	5236
Côte d'Ivoire	Lower middle	16	3418	110 006	173 553
Ghana	Lower middle	3	3511	23 785	178 321
India	Lower middle	18	100 082	3 511 214	5 082 526
Kenya	Lower middle	0	6225	0	316 137
Kyrgyzstan	Lower middle	0	669	0	33 951
Lesotho	Lower middle	0	205	0	10 408
Mauritania	Lower middle	7	573	7837	29 105
Nicaragua	Lower middle	0	570	0	28 958
Nigeria	Lower middle	17	27 327	922 743	1 387 758
Pakistan	Lower middle	25	22 940	1 164 963	1 164 963
Republic of Congo	Lower middle	0	705	0	35 787
Republic of Sudan	Lower middle	23	5322	244785	270266
Senegal	Lower middle	0	2219	0	112 695
South Sudan	Lower middle	9	1399	24665	71023
Tajikistan	Lower middle	23	1125	51 199	57 107
Tanzania	Lower middle	0	8087	0	410 661
Uzbekistan	Lower middle	5	2929	29 386	148 753
Vietnam	Lower middle	8	6530	99 216	331 607
Zambia	Lower middle	0	2506	0	127 288
Zimbabwe	Lower middle	0	1911	0	97 035
Low-income countries				2 334 238	3 725 020
Lower middle income countries				6 963 445	10 972 795
All countries				9 297 682	14 697 815

Alternatively, prices might be negotiated by country groups, such as income category if it is not possible to have country-specific pricing arrangements (policy option 3). In order to ensure providing HPV vaccination in all countries within an income group (or any group for that matter) generates a net benefit, or at minimum no net loss in overall population health, requires applying the lowest price required for HPV vaccination to be cost effective in any of the countries in the group to all countries in the group. This would be US$−1 per dose (2019 US) in low-income countries and US$0 per dose (2019 US) in lower middle-income countries. If the manufacturer and/or a global donor were to fund the difference for each country, it would cost US$14.7 billion (2019 US). More net health benefits would be generated across countries than from option 2; however, the same money could avert 70 million DALYs if spent on existing interventions in these countries instead.

The net health impact and net monetary impact of achieving the WHO recommendation of 90% coverage of HPV vaccination in all Gavi-eligible countries at current Gavi-negotiated prices (US$4.50 per dose, 2019 US), with current delivery costs and existing levels of Gavi procurement support, where many countries pay below US$4.50 per dose, are presented in table 1 (columns 3 and 4). This represents existing policy (policy option 4a) and offers positive net health impact for all but three countries, implying that at current prices and levels of Gavi support HPV vaccination generates a health benefit over and above any loss incurred as a result of the money required to fund it not being available to fund other healthcare interventions for most countries. Without the Gavi procurement support (ie, at a per dose price of US$4.50 (2019 US) for all countries, option 4b), HPV vaccination generates a net health loss in eight more countries than it would with the Gavi procurement support (see table 1 columns 5 and 6).

### Sensitivity analyses

Our analysis used a discount rate of 3% for costs and benefits following common practice. While this is in line with the WHO guidance, the guidance also recommends a sensitivity analysis where health is undiscounted but costs are discounted at 3%.^26^ The resulting net health impact estimates for this sensitivity analysis are reported against the base case in online supplemental appendix table 1 and the price reductions required are reported against the base case in online supplemental appendix table 2. The health benefits of HPV vaccination often occur in future years (eg, cancer cases are averted up to decades after the vaccine has been administered). Since greater weight is attached to future health outcomes when they are undiscounted, discounting the health benefits from HPV vaccination has the effect of reducing their net present value. Therefore, HPV vaccination appears better value when no discounting is applied to health benefits. The per dose price reduction required in order for HPV vaccination to generate zero net benefit (ie, no net loss in overall population health, but also no net benefit) in countries where it generates a net loss at average market price are also lower (and more often zero or not required at all) than when a 3% discount rate is used for health benefits. If country-specific pricing were possible, the total price reduction required (ie, for all doses for all eligible girls across all countries where a price required is required) would be less than a third of that required when a 3% discount rate is applied to health benefits. Where country income-group pricing is applied, the difference in funding required is lower at US$11.6 billion (compared with US$14.7 billion when a 3% discount rate is applied to health benefits). The price for low-income countries would be US$2 per dose and for lower middle income countries it would be US$6 per dose (2019 US$).

Our analysis uses the central estimate of the marginal productivity of each HCS from the range estimated by Ochalek *et al*.^31^ As a sensitivity analysis, we use the minimum and maximum from the ranges for each country. Using the minimum (maximum) estimate for each country will tell us the maximum (minimum) health opportunity cost expected from adopting HPV vaccination and therefore lowest (highest) estimate of net health benefit from it for each country. The results of this sensitivity analysis are reported in online supplementary appendix tables 3 and 4. Using the minimum or maximum makes little overall difference to the number of countries for which HPV vaccination would be expected to generate a net health benefit. Among low-income countries, at market price (US$25 per dose) one fewer low-income country would be estimated to have a net health benefit from adopting HPV vaccination when the minimum estimate of the marginal productivity of each HCS is used. Results do not change when current per dose procurement prices are used (as in option 4a), but when the price is US$4.50 per dose (option 4b) three additional low-income countries would expect a net health benefit when the maximum estimate of the marginal productivity of each HCS is used while one fewer would expect a net health benefit when the minimum is used (compared with the central estimate). Among lower middle income countries adopting HPV vaccination would be estimated to generate a net health benefit in one fewer (more) country when the minimum (maximum) estimate of the marginal productivity of each HCS is used at market price (US$25 per dose), but results do not change when current per dose procurement prices (as in policy option 4a) or US$4.50 per dose (policy option 4b) are used. Even when introducing HPV vaccination would result in net health benefit for the same number of countries regardless of whether the minimum, central or maximum estimate is used, the magnitude of the net health benefit differs. The estimated net health benefits (losses) are greatest (lowest) when using the maximum estimate of health opportunity cost from the range. The price reduction required for HPV vaccination to generate a net health benefit in all countries is higher (lower) when using the minimum (maximum) estimate of health opportunity costs. When the minimum estimate is used the per dose price reduction required in both low-income and lower middle income countries is US$26 (2019 US; ie, the maximum that the country that can least afford to adopt HPV vaccination can afford to pay is US$−1 per dose). When the maximum estimates of health opportunity cost are used, the price reductions required for low-income and lower middle-income countries are US$26 and US$25 (2019 US), respectively, which is the same as the base case (ie, when the central estimate is used). When country-specific pricing is used (as in policy option 2), this amounts to a total of US$8.6 billion to US$9.9 billion in funding required when the maximum and minimum estimates are used, respectively. When country income group pricing is used (as in policy option 3), this amounts to a total of US$14.5 billion to US$14.8 billion in funding required when the maximum and minimum estimates are used, respectively.

## Discussion

The analysis undertaken enables an assessment of blanket recommendations (in order to, eg, inform whether, at current prices, they would be expected to improve health in all countries); an assessment of the price reduction (if any) required for a healthcare intervention to generate at minimum no net health loss; and the value of developing new healthcare interventions.

Previous analyses have used a GDP per capita threshold to judge the cost effectiveness of adopting HPV vaccination and eliminating cervical cancer.^17 18 32^ The GDP per capita threshold originates from human capital arguments made by the WHO Commission on Macroeconomics and Health around the value of a year of life.^33^ However, WHO no longer recommends it for country evaluations on the basis that it may not reflect country priorities and decision-making processes.^14^ Estimates of the marginal productivity of HCSs reflect health opportunity cost and tend to be lower than GDP per capita, and so using a GDP per capita threshold to make decisions can lead to net health losses.^8 31^ Indeed, in practice, low-income countries' actual decisions to introduce HPV vaccination, or not, reveal an implicit cost-effectiveness threshold of 30%–35% of GDP per capita.^34^ Reassuringly, our results show that HPV vaccination remains cost effective for most countries at current Gavi negotiated prices when a threshold that reflects the health opportunity cost faced by the country is used (rather than human capital arguments about the value of health spending). Although there is uncertainty around existing estimates of the marginal productivity of HCSs, using the minimum or maximum from the range of estimates from Ochalek *et al* (2018) has little impact on the results.

A blanket recommendation to introduce HPV vaccination in LMICs would result in net health losses in most countries in the absence of Gavi support. This was analysed under the assumption that these countries would face the average market price faced by countries not eligible for Gavi support. Manufacturers might be able to price discriminate (ie, using tiered pricing policy), reducing prices for these countries. However, there is no guarantee that in the absence of pooled procurement and market shaping efforts by organisations like Gavi lower-income countries would pay lower prices.^35^ The extent to which prices would be lower than the average market price among high-income countries in the absence of Gavi support is unclear but would result in better net health impacts, while higher prices would result in worse net health impacts. It is worth noting that our estimate for policy option 1 being conservative or optimistic has no implications for comparisons between policy options 2, 3, 4a and 4b.

The current reality of vaccine procurement prices paid by countries likely falls somewhere between policy options 2 and 4. Policy option 4 assumed all countries pay S$4.50 per dose or less (as countries purchase a share of the vaccines provided while Gavi purchases the remainder). The share funded by Gavi is based on the Gavi cofinancing mechanism depending on the funding phase the country is in (based on country income level and years of funding) rather than reflecting health opportunity costs as in policy option 2.^22^ However, in practice, countries may not meet their cofinancing requirements. Gavi funding a greater proportion of vaccines would have the effect of reducing the per dose price below the prices used in 4a, and all else the same the vaccine would generate a more positive net impact in those countries. Determining prices using a more systematic method of accounting for opportunity costs, as illustrated in policy option 2, offers an opportunity to ensure that countries that do not benefit from adopting HPV vaccination at current prices would be able to introduce it without facing a reduction in population health. Future research could seek to establish the best way for donors to support the affordability of interventions, which would require information on the opportunity cost of donor financial support and the loss of revenue for the manufacturers of vaccine, in addition to the transaction costs associated with implementing donor support mechanisms.

This type of analysis can also help to inform the value of developing a new healthcare intervention that does not yet exist. For example, the Bill and Melinda Gates Foundation funds the development of new healthcare interventions targeted towards the leading causes of death and disability in LMICs. While a more uncertain prospect at this earlier stage, estimating the price at which a healthcare intervention would have a positive net health impact in each HCS (as in policy option 2) can help to inform whether it should be considered for development by the foundation, through comparison with the cost of the intervention to the provider. Using the expected net health impact of different potential healthcare interventions (and how this is distributed across countries) to rank potential investments would ensure that new healthcare interventions which are likely to generate the greatest health gains offered at affordable prices are prioritised over others. Value of information analysis provides a means to prioritise future research to resolve uncertainties with the new healthcare interventions under consideration for development. However, the development of a new healthcare intervention may be seen to address multiple objectives in addition to improving overall population health. This framework could be extended to incorporate other objectives, such as equity, following, for example, extended or distributional cost-effectiveness analysis methods. In fact, prioritisation decisions for both vaccine research and development (eg, the Vaccine Innovation Prioritisation Strategy) and financing for adoption (eg, the Gavi Investment Strategy) use a kind of multicriteria decision analysis that considers cost effectiveness alongside multiple other criteria.^36 37^

Equity concerns may also be relevant for interventions for which a net health benefit is generated in some, but not all, countries in which it is recommended. The benefit associated with providing HPV vaccination in some countries may outweigh the losses in other countries if, for example, to reflect equity being another objective in decision-making more weight is given to gains in low-income countries and this is where the bulk of the gains are. Assessing the net health impact of providing a healthcare intervention for each country enables decision-makers to be explicit about the trade-offs being made if, for example, net health losses were incurred in some low-income countries but no lower middle income countries. If more weight was put on outcomes in low-income countries, a blanket recommendation of providing the healthcare intervention would appear to be less favourable as policy choice if the price was the same in all countries as this is where a disproportionately larger amount of the overall losses in net health would be incurred.

The results are sensitive to the discount rate used. The WHO recommends initially using the discount rate used in the country, and where national guidelines do not exist recommends two scenarios: our base case (ie, 3% discounting for both health and consumption) and sensitivity analysis (ie, 3% and 0% discounting for consumption and health, respectively).^26^ However, application of the same discount rates for all countries is unlikely to be appropriate. Where the objective is improving population health, for example, the appropriate discount rate for health for each country should depend on the rate at which the principal can borrow and save and the expected growth in the marginal productivity of the HCS^38 39^—both of which would be expected to vary by country. To date, there are no data on the expected growth in the marginal productivity of the HCS, and this should be a priority for future research.

## Conclusion

This paper illustrates how estimates of the net health impact or, equivalently, net monetary value of providing a healthcare intervention can be used to estimate the expected effect to overall population health in a country of adhering to global guidance and inform negotiations with donors, as well as informing pricing negotiations and the value of developing new healthcare interventions. At Gavi negotiated prices, HPV vaccination generates net health benefits across nearly all Gavi-eligible countries included in this study. However, if Gavi-eligible countries faced the same price as non-Gavi eligible countries, providing HPV vaccination would reduce overall population health in all but two low-income and nearly half of lower middle income countries and impose a net DALY burden of 38 million DALYs globally. This suggests that Gavi’s negotiations on behalf of countries eligible for its support have succeeded in making adhering to the WHO guidance around HPV vaccination a worthy aim for most countries. Assessing the likely scale and distribution of the impact of potential new interventions on net population health, at a particular price, can also inform priorities in developing new technologies. The maximum price each country could afford to pay for HPV vaccination to be cost effective can be calculated as can the net health benefit that would be expected to be generated at different potential prices. Determining prices using these methods, which account for country-specific opportunity costs, offers an opportunity to ensure that all countries can benefit from adopting HPV vaccination or other recommendations made by global bodies. Finally, the methods used here can be applied to assess the value of developing a new technology. It will also depend on not only the expected costs and benefits of the new technology for each country in which it may be implemented, but as well the likely health opportunity costs in those countries.
