# Supplementary material for: Assessing the value of human papillomavirus vaccination in Gavi-eligible low-income and middle-income countries
Source: BMJ Glob Health. 2020 Oct 20;5(10):e003006. doi: 10.1136/bmjgh-2020-003006 (PMC7577028; doi:10.1136/bmjgh-2020-003006)
Supplement: Supplementary data [file bmjgh-2020-003006supp001.pdf]

Supplementary Appendix 1. Calculated per dose price

Estimates of the additional cost of HPV vaccination reflect the three phases of the Gavi co-financing mechanism: 1) initial self-financing, 2) preparatory transition, and 3) accelerated transition. Countries in the first phase co-finance a portion of the HPV vaccines equivalent to $0.20 per dose (or 4.4% of the cost of a dose). Countries remain in the first phase until their gross national income (GNI) per capita exceeds $1,005.

Countries with a GNI per capita above $1,005 and up to $1,580 per capita are in the second phase (i.e., the preparatory transition phase), and pay 15% more for each dose each year. If HPV vaccination is introduced when countries are already in this phase, we calculate the price per dose as:

$$HPV\ vaccine\ price\ per\ dose\ (phase\ 2) = \$4.50 - \frac{\$0.20}{\$4.50} * 1.15^{t}$$

where *t* is the number of years the country has been in the preparatory transition phase.

Countries move from the second transition phase into the third phase once their GNI per capita is above $1,580, and can remain in this phase for five years, at which point they graduate from Gavi funding to become fully self-financing. For countries in the first year of this phase in 2020, price per dose increases 15% and is calculated as:

$$HPV\ vaccine\ price\ per\ dose\ (phase\ 3\ year\ 1\ of\ 5) = \$4.50 - \frac{\$0.20}{\$4.50} * 1.15^{t+1}$$

In the following four years, the co-financed share increases linearly to reach $4.50.

Supplementary Appendix Table 1. Sensitivity analysis around discount rates: Net health impact (NHI) and net monetary impact (NMI)

A: Base case (discount rate of 3% for health and 3% for costs)

B: Discount rate of 0% for health and 3% for costs

	$25 per dose		Gavi procurement support (per dose price differs by country, all <=$4.50)		$4.50 per dose	
	A	B	A	B	A	B
**Low-income**						
Net health benefits						
# of countries	2	13	21	22	15	21
Total +ive NHI (1,000s)	201	16,104	8,766	35,646	5,203	31,262
Total +ive NMI (1,000s 2019 US$)	33,281	2,671,456	1,327,194	5,490,501	837,388	4,884,613
Net health losses						
# of countries	20	9	1	0	7	1
Total -ive NHI (1,000s)	-17,134	-6,118	-37	0	-909	-50
Total -ive NMI (1,000s 2019 US$)	-2,331,849	-797,328	-9,388	0	-138,035	-12,565
Net health impact						
# of countries	22	22	22	22	22	22
Total NHI (1,000s)	-16,932	9,986	8,728	35,646	4,294	31,212
Total NMI (1,000s 2019 US$)	-2,298,568	1,874,127	1,317,805	5,490,501	699,353	4,872,048
**Lower middle-income**						
Net health benefits						
# of countries	12	20	22	24	19	24
Total +ive NHI (1,000s)	1,781	28,844	9,646	53,350	8,112	50,594
Total +ive NMI (1,000s 2019 US$)	819,677	12,087,170	3,609,966	19,502,849	3,181,013	18,879,844
Net health losses						
# of countries	12	4	2	0	5	0
Total -ive NHI (1,000s)	-23,277	-6,033	-216	0	-1,501	0
Total -ive NMI (1,000s 2019 US$)	-5,798,249	-982,153	-43,809	0	-262,383	0
Net health impact						
# of countries	24	24	24	24	24	24

Total NHI (1,000s)	-21,496	22,811	9,431	53,350	6,610	50,594
Total NMI (1,000s 2019 US$)	-4,978,573	11,105,017	3,566,157	19,502,849	2,918,630	18,879,844
**All countries**						
Net health benefits						
# of countries	14	33	43	46	34	45
Total +ive NHI (1,000s)	1,983	44,948	18,412	88,996	13,315	81,856
Total +ive NMI (1,000s 2019 US$)	852,957	14,804,203	4,913,255	24,791,372	4,027,366	23,641,054
Net health losses						
# of countries	32	13	3	0	12	1
Total -ive NHI (1,000s)	-40,411	-12,151	-253	0	-2,410	-50
Total -ive NMI (1,000s 2019 US$)	-7,672,817	-1,545,850	-53,198	0	-354,714	-12,565
Net health impact						
# of countries	46	46	46	46	46	46
Total NHI (1,000s)	-38,428	32,797	18,159	88,996	10,904	81,806
Total NMI (1,000s 2019 US$)	-6,819,860	13,258,353	4,860,057	24,791,372	3,672,652	23,628,489

Supplementary Appendix Table 2. Sensitivity analysis around discount rates: Pricing arrangements

A: Base case (discount rate of 3% for health and 3% for costs)

B: Discount rate of 0% for health and 3% for costs

Country	Income group	Per dose price reduction required (2019 US$)		Total reduction using country specific price (1,000s 2019 US$, option 2)		Total reduction using country income group price (1,000s, $2 for low-income countries, $6 for lower middle-income countries, 2019 US$, option 3)	
		A	B	A	B	A	B
Benin	Low	18	0	57,749	0	80,661	72,268
Burkina Faso	Low	10	0	57,074	0	146,744	131,475
Burundi	Low	9	0	31,075	0	87,647	78,527
Chad	Low	21	8	101,129	37,013	122,104	109,399
Democratic Republic of the Congo	Low	23	14	582,915	348,943	663,039	594,048
Eritrea	Low	22	8	18,430	6,879	21,877	19,600
Ethiopia	Low	17	0	491,992	0	725,995	650,454
Gambia	Low	9	0	5,981	0	17,090	15,312
Guinea	Low	14	0	48,351	0	89,790	80,448
Guinea Bissau	Low	22	11	11,104	5,759	13,159	11,790
Haiti	Low	21	3	45,173	7,576	55,990	50,164
Madagascar	Low	15	0	105,294	0	176,781	158,386
Malawi	Low	0	0	0	0	129,186	115,744
Mali	Low	19	1	108,948	6,179	150,676	134,998
Mozambique	Low	2	0	20,123	0	222,326	199,193
Nepal	Low	13	0	62,126	0	124,454	111,505
Niger	Low	24	18	183,674	134,466	196,160	175,749
Rwanda	Low	8	0	25,035	0	81,666	73,168

Sierra Leone	Low	24	16	46,162	30,374	50,321	45,085
Togo	Low	21	4	42,623	8,095	53,232	47,693
Uganda	Low	8	0	108,355	0	335,200	300,322
Yemen	Low	26	23	180,923	162,097	180,923	162,097
Bangladesh	Lower middle	23	13	592,799	321,937	645,928	483,731
Cambodia	Lower middle	20	0	61,602	0	78,677	58,921
Cameroon	Lower middle	17	0	119,244	0	175,050	131,094
Comoros	Lower middle	0	0	0	0	5,236	3,921
Côte d'Ivoire	Lower middle	16	0	110,006	0	173,553	129,972
Ghana	Lower middle	3	0	23,785	0	178,321	133,543
India	Lower middle	18	0	3,511,214	0	5,082,526	3,806,270
Kenya	Lower middle	0	0	0	0	316,137	236,753
Kyrgyzstan	Lower middle	0	0	0	0	33,951	25,426
Lesotho	Lower middle	0	0	0	0	10,408	7,795
Mauritania	Lower middle	7	0	7,837	0	29,105	21,796
Nicaragua	Lower middle	0	0	0	0	28,958	21,686
Nigeria	Lower middle	17	0	922,743	0	1,387,758	1,039,283
Pakistan	Lower middle	25	19	1,164,963	872,433	1,164,963	872,433
Republic of Congo	Lower middle	0	0	0	0	35,787	26,801
Republic of Sudan	Lower middle	23	6	244,785	66,516	270.266	202,400
Senegal	Lower middle	0	0	0	0	112,695	84,396
South Sudan	Lower middle	9	0	24,665	0	71,023	53,189
Tajikistan	Lower middle	23	9	51,199	21,121	57,107	42,767
Tanzania	Lower middle	0	0	0	0	410,661	307,542
Uzbekistan	Lower middle	5	0	29,386	0	148,753	111,400
Vietnam	Lower middle	8	0	99,216	0	331,607	248,338
Zambia	Lower middle	0	0	0	0	127,288	95,325

Zimbabwe	Lower middle	0	0	0	0	97,035	72,669
**Low**				2,334,238	747,380	3,725,020	3,337,425
**Lower middle**				6,963,445	1,282,007	10,972,795	8,217,453
**All**				9,297,682	2,029,387	14,697,815	11,554,878

Supplementary Appendix Table 3. Sensitivity analysis around estimate of health opportunity cost: Net health impact (NHI) and net monetary impact (NMI)

	$25 per dose (option 1)			Gavi procurement support (per dose price differs by country, all <=$4.50, option 4a)			$4.50 per dose (option 4b)		
	Minimum	Central estimate of health opportunity cost	Maximum	Minimum	Central estimate of health opportunity cost	Maximum	Minimum	Central estimate of health opportunity cost	Maximum
**Low-income**									
Net health benefits									
# of countries	1	2	2	21	21	21	14	15	18
Total +ive NHI (1,000s)	94	201	375	8,835	8,766	8,692	4,982	5,203	5,459
Total +ive NMI (1,000s 2019 US$)	12,603	33,281	76,156	1,168,107	1,327,194	1,539,249	704,538	837,388	1,017,070
Net health losses									
# of countries	21	20	20	1	1	1	8	7	4
Total -ive NHI (1,000s)	-20,107	-17,134	-13,679	-47	-37	-30	-1,171	-909	-592
Total -ive NMI (1,000s 2019 US$)	-2,471,097	-2,331,849	-2,161,792	-10,228	-9,388	-8,511	-165,112	-138,035	-104,785
Net health impact									
# of countries	22	22	22	22	22	22	22	22	22
Total NHI (1,000s)	-20,013	-16,932	-13,304	8,787	8,728	8,662	3,811	4,294	4,866
Total NMI (1,000s 2019 US$)	-2,458,495	-2,298,568	-2,085,635	1,157,879	1,317,805	1,530,738	539,426	699,353	912,286
**Lower middle-income**									
Net health benefits									
# of countries	10	11	12	22	22	22	20	20	20
Total +ive NHI (1,000s)	1,427	1,715	1,988	9,306	9,714	10,060	7,641	8,185	8,653
Total +ive NMI (1,000s 2019 US$)	564,543	797,406	1,093,574	2,967,884	3,635,420	4,388,940	2,557,94	3,209,078	3,943,923
Net health losses									
# of countries	14	13	12	2	2	2	4	4	4
Total -ive NHI (1,000s)	-28,007	-22,755	-18,118	-345	-	-93	-1,889	-1,442	-1,046

Total -ive NMI (1,000s 2019 US$)	-6,055,675	-5,603,628	-5,124,730	-61,184	-43,809	-22,264	-274,246	-240,472	-200,251
Net health impact									
# of countries	24	24	24	24	24	24	24	24	24
Total NHI (1,000s)	-26,580	-21,041	-16,130	8,961	9,498	9,967	5,752	6,743	7,607
Total NMI (1,000s 2019 US$)	-5,491,133	-4,806,222	-4,031,156	2,906,700	3,591,610	4,366,676	2,283,69	2,968,606	3,743,672
**All countries**									
Net health benefits									
# of countries	11	13	14	43	43	43	34	35	38
Total +ive NHI (1,000s)	1,521	1,916	2,363	18,140	18,479	18,752	12,623	13,388	14,111
Total +ive NMI (1,000s 2019 US$)	577,145	830,687	1,169,730	4,123,966	4,938,709	5,890,201	3,268,65	4,055,431	4,972,751
Net health losses									
# of countries	35	33	32	3	3	3	12	11	8
Total -ive NHI (1,000s)	-48,114	-39,889	-31,797	-392	-	-	-3,060	-2,351	-1,638
Total -ive NMI (1,000s 2019 US$)	-8,057,611	-7,478,196	-6,843,323	-71,412	-53,198	-30,775	-378,979	-332,803	-276,208
Net health impact									
# of countries	46	46	46	46	46	46	46	46	46
Total NHI (1,000s)	-46,593	-37,973	-29,434	17,748	18,226	18,629	9,563	11,036	12,473
Total NMI (1,000s 2019 US$)	-7,480,465	-6,647,509	-5,673,593	4,052,554	4,885,511	5,859,426	2,889,67	3,722,627	4,696,543

Supplementary Appendix Table 4. Sensitivity analysis around estimate of health opportunity cost: Pricing arrangements

Country	Income group	Per dose price reduction required (2019 US$)			Total reduction using country specific price (1,000s 2019 US$, option 3)			Total reduction using country income group price (1,000s 2019 US, option 4)		
Estimate of health opportunity cost		Min.	Central	Max.	Minimum	Central	Maximum	Minimum ($-1 for low-income countries, $-1 for lower-middle income countries)	Central ($-1 for low-income countries, $0 for lower-middle income countries)	Maximum ($-1 for low-income countries, $0 for lower-middle income countries)
Benin	Low	19	18	18	60,544	57,749	54,956	81,035	80,661	80,269
Burkina Faso	Low	11	10	7	63,934	57,074	41,470	147,426	146,744	146,033
Burundi	Low	11	9	7	36,106	31,075	22,783	88,054	87,647	87,222
Chad	Low	22	21	20	103,060	101,129	96,947	122,671	122,104	121,512
Democratic Republic of the Congo	Low	23	23	22	589,252	582,915	572,297	666,116	663,039	659,822
Eritrea	Low	22	22	21	18,858	18,430	17,886	21,978	21,877	21,770
Ethiopia	Low	18	17	16	519,696	491,992	455,269	729,365	725,995	722,473
Gambia	Low	11	9	7	7,359	5,981	4,424	17,170	17,090	17,008
Guinea	Low	15	14	12	51,670	48,351	42,168	90,207	89,790	89,355
Guinea Bissau	Low	22	22	21	11,246	11,104	10,826	13,220	13,159	13,096
Haiti	Low	22	21	20	47,084	45,173	43,511	56,250	55,990	55,718
Madagascar	Low	16	15	14	111,744	105,294	97,157	177,601	176,781	175,923
Malawi	Low	1	0	0	4,039	0	0	129,786	129,186	128,559
Mali	Low	19	19	17	111,987	108,948	102,302	151,376	150,676	149,946
Mozambique	Low	5	2	0	44,948	20,123	0	223,358	222,326	221,247
Nepal	Low	15	13	11	73,030	62,126	52,890	125,032	124,454	123,851

Niger	Low	24	24	24	185,145	183,674	180,348	197,070	196,160	195,208
Rwanda	Low	10	8	6	32,167	25,035	17,935	82,045	81,666	81,269
Sierra Leone	Low	24	24	23	46,581	46,162	45,308	50,554	50,321	50,076
Togo	Low	21	21	20	43,783	42,623	41,179	53,479	53,232	52,974
Uganda	Low	10	8	6	130,674	108,355	78,439	336,756	335,200	333,574
Yemen	Low	26	26	26	181,762	180,923	180,045	181,762	180,923	180,045
Bangladesh	Lower middle	24	23	23	607,475	592,799	575,924	652,287	645,928	637,843
Cambodia	Lower middle	21	20	19	65,569	61,602	58,604	79,452	78,677	77,693
Cameroon	Lower middle	18	17	16	123,380	119,244	112,024	176,774	175,050	172,859
Comoros	Lower middle	0	0	0	0	0	0	5,288	5,236	5,170
Côte d'Ivoire	Lower middle	17	16	15	115,698	110,006	100,313	175,261	173,553	171,380
Ghana	Lower middle	6	3	1	42,040	23,785	3,723	180,076	178,321	176,089
India	Lower middle	19	18	16	3,761,298	3,511,214	3,239,681	5,132,563	5,082,526	5,018,906
Kenya	Lower middle	0	0	0	0	0	0	319,250	316,137	312,180
Kyrgyzstan	Lower middle	0	0	0	0	0	0	34,285	33,951	33,526
Lesotho	Lower middle	0	0	0	0	0	0	10,511	10,408	10,278
Mauritania	Lower middle	9	7	4	10,097	7,837	5,041	29,391	29,105	28,741
Nicaragua	Lower middle	0	0	0	0	0	0	29,243	28,958	28,595
Nigeria	Lower middle	18	17	15	970,840	922,743	838,130	1,401,421	1,387,758	1,370,387
Pakistan	Lower middle	26	25	25	1,176,432	1,164,963	1,150,381	1,176,432	1,164,963	1,150,381
Republic of Congo	Lower middle	0	0	0	0	0	0	36,140	35,787	35,339
Republic of Sudan	Lower middle	24	23	22	250,691	244,785	237,822	272,927	270,266	266,883
Senegal	Lower middle	1	0	0	3,988	0	0	113,804	112,695	111,284
South Sudan	Lower middle	11	9	7	30,157	24,665	18,190	71,723	71,023	70,134
Tajikistan	Lower middle	24	23	22	52,923	51,199	49,400	57,670	57,107	56,392
Tanzania	Lower middle	0	0	0	0	0	0	414,704	410,661	405,521

Uzbekistan	Lower middle	9	5	1	53,265	29,386	7,204	150,218	148,753	146,891
Vietnam	Lower middle	12	8	4	156,971	99,216	54,461	334,872	331,607	327,456
Zambia	Lower middle	0	0	0	0	0	0	128,541	127,288	125,695
Zimbabwe	Lower middle	0	0	0	0	0	0	97,990	97,035	95,820
**Low**					2,474,668	2,334,238	2,158,140	3,742,310	3,725,020	3,706,950
**Lower-middle**					7,420,824	6,963,445	6,450,897	11,080,821	10,972,795	10,835,444
**All**					9,895,492	9,297,682	8,609,037	14,823,131	14,697,815	14,542,394
